# Cytotoxic Activity of Six Samples of Brazilian Propolis on Sea Urchin (*Lytechinus variegatus*) Eggs

**DOI:** 10.1155/2013/619361

**Published:** 2013-04-09

**Authors:** C. C. Fernandes-Silva, J. C. Freitas, A. Salatino, M. L. F. Salatino

**Affiliations:** ^1^Department of Botany, Institute of Biosciences, University of São Paulo, Rua do Matão, 277, 05508-090 São Paulo, Brazil; ^2^Department of Human and Comparative Physiology, Institute of Biosciences, University of São Paulo, Rua do Matão, 101, 05508-900 São Paulo, Brazil

## Abstract

The cytotoxic activities of extracts of four samples of propolis from the state of Minas Gerais (Southeast Brazil) and two from the state of Paraná (South Brazil) were evaluated using sea urchin (*Lytechinus variegatus*) eggs. Cytotoxic activity was observed, characterized mainly by the inhibition of the first cleavage of newly fertilized eggs. Methanol extracts at 32 *µ*g mL^−1^ of all samples were highly active (97–100%). Extracts were also prepared by successive treatments of the samples with hexane, chloroform, ethyl acetate, and methanol. High activity was observed using the ethyl acetate fractions of all samples, but hexane and chloroform fractions of some samples also had high activity. Based on the chemical composition of the extracts and fractions (published previously), it is hypothesized that the cytotoxic activities observed are due mainly to artepillin C, *p*-coumaric acid, and kaempferide. The results suggest that caffeoylquinic acids have no cytotoxic activity in sea urchin eggs.

## 1. Introduction

The cure of cancer is still a major challenge and the study of antitumoral compounds is of great importance in the search for new drugs and treatments. Several natural products, including propolis, have shown high cytotoxic and antitumoral activities. Propolis is a complex mixture of substances with resinous aspect, prepared mostly by *Apis mellifera* honeybees from plant exudates and beeswax. Its chemical composition varies widely, according to the flora around the hive. In Brazil the main source of the production of propolis is *Baccharis dracunculifolia* (alecrim-do-campo, Asteraceae). Among Brazilian propolis, the green type is the most commercialized and exported, chiefly to Japan. Its main constituents are prenylated phenylpropanoids and caffeoylquinic acids [1]. 

The number of studies about antitumor activity of propolis extracts and isolated compounds has increased in recent years. The results have shown that several tumor cell lines are sensitive to propolis. Green propolis water soluble derivatives suppressed the development of metastasis of lung tumors in mice [2]. Water extracts were also effective at inhibiting the growth of mouse sarcoma and reveled a significant reduction in mitotic cells and tumor invasion [3]. Ethanol extracts of Brazilian green propolis inhibit the proliferation of prostate cancer cells in a dose-dependent manner [4] and inhibit also the growth of colon cancer cells [5]. Prenylated phenylpropanoids, such as artepillin C, and flavonoids isolated from Brazilian propolis possess antitumoral activity [6]. Cinnamic acid derivatives isolated from Brazilian propolis, such as drupanin and baccharin, showed antitumor effects on murine fibrosarcoma [7]. 

Results of analyses by GC/MS and HPLC/DAD/ESI/MS/MS of four samples of propolis from the state of Minas Gerais (MG, Southeast Brazil) and two from the state of Paraná (PR, South Brazil) were published recently [8]. The samples from the two states differed regarding the exclusive presence of luteolin-5-*O*-methyl ether in the samples from Paraná, a region on the border of the distribution of *Baccharis dracunculifolia*. Caffeoylquinic acids were abundant in all samples, but kaempferide, isorhamnetin, and *p-*coumaric acid were more abundant in samples from Minas Gerais. Prenylated phenylpropanoids, such as artepillin C, chromanes, and baccharin were also detected, more abundantly in the samples from Minas Gerais.

The analysis of alterations in the development of sea urchin (*Lytechinus variegates*) eggs has been regarded as a suitable model for evaluating cytotoxic, antiproliferative, and other biological activities [9–13]. The aim of the present work was to determine the cytotoxic activity of extracts of the above mentioned samples of Brazilian propolis. To our knowledge, this is the first study using sea urchin eggs in studies about cytotoxic activity of propolis.

## 2. Material and Methods

### 2.1. Propolis Samples and Extraction

Six samples of Brazilian green propolis produced by Africanized *Apis mellifera* L. were analyzed. The samples stemmed from the states of MG (samples A-C: municipality of Esmeraldas: 19°22′46′′ S, 44°18′47′′ W; sample D: municipality of Três Pontas: 21°22′00′′ S, 45°18′45′′ W) and PR (samples E and F: municipality of União da Vitória: 26°13′54′′ S, 51°04′08′′ W) were analyzed. Powdered portions of 2.5 g of each sample of propolis were extracted with methanol for 6 h in Soxhlet. In parallel, powdered portions of 5 g of each sample of propolis were treated successively with solvents of increasing polarity in Soxhlet for 6 h with each solvent. For the sake of convenience, the product of the first extraction is called “methanol extract” and the other products are referred to as “fractions” (hexane, chloroform, ethyl acetate, and methanol fractions). The methanol extract and all fractions were concentrated under reduced pressure and dissolved in ethanol for analysis. Chemical composition of the six samples are published elsewhere [8].

### 2.2. Determination of Cytotoxic Activity

Antimitotic activity was assumed as the ability of extracts to inhibit the cleavage of sea urchin eggs. The elimination of gametes was induced by injection of 0.5 M KCl in the perivisceral cavity. The tests were performed on plates of 12 wells (Corning) by mixing 1 mL of the sperm suspension (0.1 mL of sperm + 4.9 mL of filtered seawater) with 20 *μ*L of eggs. Two minutes after fertilization, 10 *μ*L of ethanol solutions of the extracts was added, plus filtered sea water to make up the volume of 2 mL. As control, 10 *μ*L of ethanol was used. For establishment of the concentration of the methanol extract and fractions to be used, solutions of the MeOH extract were prepared at concentrations 8, 16, and 32 *μ*g mL^−1^. At 32 *μ*g mL^−1^ nearly all embryos were affected; hence all extracts and fractions were diluted at this concentration for determination of cytotoxic activity. The plates were kept at room temperature (26 ± 2°C). At appropriate intervals, when most embryos were in the second and third cleavages (four and eight cells), aliquots of 500 *μ*L were fixed in 4% formaldehyde for detailed observation. One hundred eggs or embryos were observed in triplicate for each extract and the number of embryos with normal development was counted. An Olympus microscope model CBA was used and images were obtained with a digital camera Canon PowerShot A520. All tests were carried out in three pseudoreplicates of the same sample and the results are presented as mean ± standard deviation.

## 3. Results and Discussion

**Figure 1:**
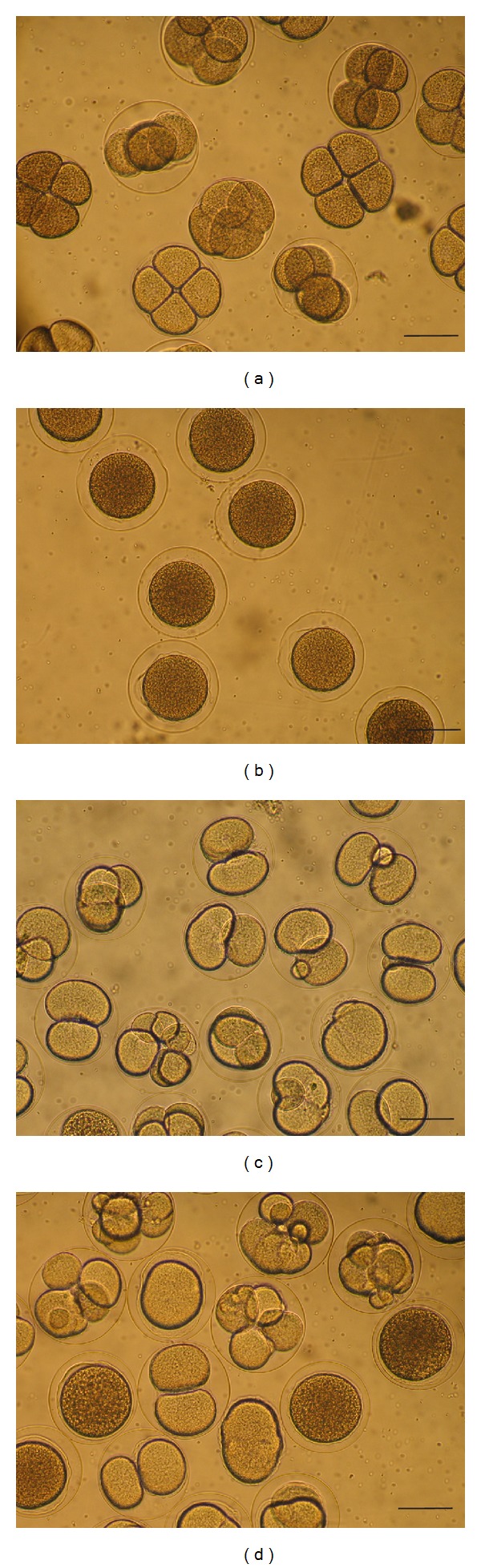
Micrographs evidencing the effect of methanol extract and fractions of samples of Brazilian propolis at 32 *μ*g mL^−1^ on the initial development of sea urchin (*Lytechinus variegatus*) embryos. (a) control (ethanol): normal development; (b) effect of methanol extract of sample D: inhibition of egg cleavage; (c) effect of chloroform fraction of sample D: eggs with abnormal division; (d) effect of ethyl acetate fraction of sample A: eggs with abnormal division or inhibition of cleavage. A and D: samples from Minas Gerais state. For detailed information regarding the activity of extracts of all samples and extracts, see Table 1. Scale bars = 100 *μ*m.

Cytotoxic activity was often observed, consisting on the inhibition of the first cleavage of newly fertilized eggs, which is a characteristic antimitotic effect (Figure 1). In addition to the inhibition of egg cleavage, in some cases abnormalities of egg development were also observed. Figure 1 depicts patterns of inactivity or normal cleavage (a, exemplified by the control ethanol alone), total inhibition of egg cleavage (b, exemplified by methanol extract of sample D), abnormalities of egg development (c, exemplified by chloroform fraction of sample D), and inhibition of egg cleavage together with abnormal egg development (d, exemplified by ethyl acetate fraction of sample A). Similar patterns of cytotoxic activity and inactivity were observed with extracts not represented in Figure 1. Complete inactivity on fertilized eggs was observed in tests with the control (ethanol). Results of inhibition observed in tests of all extracts and fractions are given in Table 1. The cytotoxicity induced by extracts or fractions of the propolis samples may be related to the inhibition of DNA replication and/or synthesis of proteins [11], suggesting the presence of substances with cytotoxic activity in the analyzed propolis samples. 

The average number of normal embryos after treatment of eggs with the methanol extract of sample B at 8, 16, and 32 *μ*g mL^−1^ was 4.7%  ± 1.2, 34.3%  ± 6.6, and 97.3%  ± 2.1 (mean ± s.d.), respectively. Solutions at 32 *μ*g mL^−1^ of the methanol extract of all samples exerted high cytotoxic activity, inhibiting nearly 100% of the cleavages (Table 1). Differences among samples and extracts were observed. The activities of the hexane fractions at 32 *μ*g mL^−1^ varied among the samples A–F, ranging from 4.3% (sample F, PR) to 98.5% (sample A, MG; Table 1). The chloroform fractions of the samples from PR (E and F) showed low cytotoxic activity (2% and 15%, resp.), while their counterparts from MG exerted medium or high activity (A and B: 83%; C: 64%; D: 92.5%; Table 1). It is remarkable that the ethyl acetate fraction from all samples (A–F) exhibited high cytotoxic activity (95.5–100%; Table 1). On the other hand, the remaining constituents extracted with methanol, after removal of substances by treatments with hexane, chloroform, and ethyl acetate, exerted low activity (1.7–4.7%, Table 1).

Major constituents of the ethyl acetate fractions from MG (A–D), detected by HPLC/DAD/ESI/MS/MS analysis, were *p-*coumaric acid, methoxypinobanksin, isorhamnetin, and kaempferide; in samples from PR (E and F) the major constituents were *p*-coumaric acid, 3-prenyl-4-(2-methylpropionyloxy)-cinnamic acid, and luteolin-5-methyl-ester [8]. Drupanin, 3-hydroxy-2,2-dimethyl-8-prenylchromane-6-propenoic acid, and artepillin C (a marker substance of Brazilian green propolis) were also detected in the ethyl acetate fraction of all samples, although not among the major constituents [8]. Previous studies have shown that *p*-coumaric acid possesses antiproliferative effect on colon cancer cells [14, 15]. Artepillin C suppresses angiogenesis induced by tumors [16] and inhibits the growth of neurofibromatosis tumors [17]. Kaempferide inhibits hypoxia-inducible factor (HIF)-1, a key mediator in tumor adaptation and survival [18]. These compounds, alone or in synergism, could account for the cytotoxic effects observed in the present study. In the hexane and chloroform fractions of all studied samples, major compounds detected by GC/EIMS analysis were artepillin C, benzenepropanoic acid, and 3-prenylcinnamic acid allyl ester [8]. Only samples from MG (A and B: hexane fraction; A–D: chloroform fraction) revealed relevant cytotoxic activity. A hypothesis may be raised that a crucial substance for the observed activity is artepillin C. 3-Prennylcinnamic acid allyl ester has never been tested for cytotoxic and antitumoral activities. Caffeoylquinic acids are major compounds in the methanol fraction of the six studied samples. Since these fractions practically showed no cytotoxic activity (Table 1), a conclusion may be drawn that caffeoylquinic acids are ineffective at suppressing the cleavage of sea urchin eggs or the development of the embryos. However, these compounds have been shown to induce apoptosis on HL-60 cells [7] and to possess antimutagenic effects [19]. These observations strengthen the importance of taking into account results from distinct models of testing cytotoxic and antiproliferative activity. 

## 4. Conclusion

Cytotoxicity is a biological activity very common among samples of propolis from diverse areas of the Brazilian territory. The most effective constituents of Brazilian green propolis seem to have medium polarity, since higher activity was observed in tests with the ethyl acetate fraction. Results of the present work, combined with data from chemical analysis of the same propolis samples, indicate that high polar propolis constituents, such as caffeoylquinic acids, seem to be ineffective against *Lytechinus variegatus *eggs. The study of the development of sea urchin embryos is a practical and efficient model to evaluate the cytotoxicity of extracts and pure compounds and is thus qualified as a useful method in the search of propolis constituents with antitumoral potential.

## Figures and Tables

**Table 1:** Cytotoxic activity, (∗) expressed as percent of affected sea urchin (*Lytechinus variegatus*) embryos, of methanol extract (MeOH) and hexane, chloroform (CHCl_3_), ethyl acetate (EtOA), and methanol (MeOH) fractions at 32 *µ*g mL^−1^ (see text) from samples (∗∗) of Brazilian propolis. No effect was observed in tests using ethanol (control).

Extract/fractions (*µ*g/mL)			Samples			
	A	B	C	D	E	F
MeOH extract	98.5 ± 2.1	97.3 ± 2.1	100 ± 0.0	99.3 ± 0.6	97.7 ± 3.2	97.3 ± 1.5
Fractions						
Hexane	98.5 ± 2.1	64.7 ± 7.5	27.5 ± 0.7	5.0 ± 2.1	4.3 ± 2.1	6.3 ± 1.5
CHCl_3_	83.0 ± 1.4	83.3 ± 7.5	64.0 ± 4.2	92.5 ± 0.7	2.0 ± 1.0	15.0 ± 1.4
EtOAc	95.5 ± 2.1	98.0 ± 2.0	100 ± 0.0	100 ± 0.0	99.7 ± 0.6	100 ± 0.0
MeOH	3.5 ± 2.1	2.3 ± 1.2	3.3 ± 0.6	3.5 ± 0.7	4.7 ± 2.5	1.7 ± 1.2

*Mean ± s.d.; **A–D: samples from Minas Gerais, E and F: samples from Paraná state.
